# Human BKV large T genome detection in prostate cancer and benign prostatic hyperplasia tissue samples by nested PCR: A case-control study

**DOI:** 10.22099/mbrc.2023.47537.1836

**Published:** 2023

**Authors:** Narges Tavassoli, Arastoo Vojdani, Sara Salimi-Namin, Majid Khadem-Rezaiyan, Mahmoudreza Kalantari, Masoud Youssefi

**Affiliations:** 1 Department of Microbiology and Virology, Faculty of Medicine, Mashhad University of Medical Sciences, Mashhad, Iran; 2 Department of Community Medicine, Faculty of Medicine, Mashhad University of Medical Sciences, Mashhad, Iran; 3 Department of Pathology, Mashhad Faculty of Medicine, Mashhad University of Medical Sciences, Mashhad, Iran; 4 Antimicrobial Resistance Research Center, Mashhad University of Medical Sciences, Mashhad, Iran; † Contributed equally as first authors

**Keywords:** BK Virus, Prostate Cancer, Benign Prostatic Hyperplasia, Nested PCR

## Abstract

Human BK polyomavirus (BKPyV) is a latent infectious agent in the genitourinary tract associated with hemorrhagic cystitis and nephropathy. This virus can be a risk factor for various human malignancies, including prostate cancer (PCa). It may contribute to prostate cancer development, as it demonstrates oncogenic properties by encoding oncoproteins. This study assessed the prevalence of this virus in benign and malignant prostate tissues. Between 2009 and 2019, 49 formalin-fixed paraffin-embedded (FFPE) PCa and 49 benign prostatic hyperplasia (BPH) samples were gathered from the pathology department of a tertiary care university hospital. They were used as cases and controls, respectively. After deparaffinization and DNA extraction, nested PCR was applied to identify the BKPyVgp5 gene (LTAg) using inner and outer primers. The nested PCR showed a 278-bp bond corresponding to the BKPyVgp5 genome (LTAg) in 53.1% (26/49) of PCa and 14.3% (7/49) of BPH (p<0.001). The presence of BKV was significantly associated with an increased risk of PCa development (OR=6.78, 95% CI=2.55–18.02, p<0.001). The BKV LTAg gene was significantly more prevalent in PCa samples than in BPH samples. These results demonstrate the presence of the virus in prostate cancer tissues.

## INTRODUCTION

As the second most common cancer in males, prostate cancer (PCa) accounted for more than 1.4 million new cancer cases worldwide in 2020 [1]. The American Cancer Society estimated more than 260,000 new prostate cancer diagnoses and more than 34,000 fatalities in 2022 [2]. Prostate cancer incidence rates vary significantly worldwide [3]. More than half of newly diagnosed cases are among men living in developed countries [4]. In Iran, prostate cancer ranks third among male malignancies [5]. Additionally, new research indicates an increase in the prevalence of prostate cancer in Asian nations like Iran and Turkey [6]. Age, race, ethnicity, and diet are risk factors for prostate cancer [7]. Benign prostatic hyperplasia (BPH) most commonly affects older adults and causes the prostate gland to grow larger than usual. Although the prostate gland is not malignant in BPH, the person experiences symptoms, including frequent urination [8, 9]. 

Researches show that infectious agents, including viruses and bacteria, are responsible for one-fifth of all incidences of cancer worldwide [10]. The effects of viruses on human cancers have attracted scientific attention [11]. Various DNA and RNA viruses, such as papillomaviruses for cervical cancer and HTLV1 for adult T-cell leukemia, have been introduced as the cause of malignancies in humans [12]. The *Polyomaviridae* family is also thought to contribute to human cancer [13]. Studies provided supporting evidence on the possible role of JC and BKV polyomavirus infection in developing colorectal cancer [14] and MCV polyomavirus infection in bladder cancer [15]. Particularly in prostate cancer, exposure to infectious agents, such as viruses, causing inflammation, has been introduced as a possible cause of prostate epithelial tissue damage and, eventually, prostate cancer [7]. 

The human BK Virus (BKV) belongs to the *Polyomaviridae* family. It is a small, non-enveloped virus with an icosahedral-shaped capsid and circular double-stranded DNA [16]. Serological investigations reveal that antibodies against this virus are found in almost all human populations [17]. It primarily infects the renal system, lying dormant in healthy individuals but reactivating in immunocompromised patients [18]. Animal studies conducted in vivo and/or in vitro provide evidence of BKV carcinogenic potential. Consequently, the World Health Organization (WHO) classifies it as "possibly carcinogenic to humans" [19].

 The BKV genome consists of early and late genomic sections that encode functional and structural proteins, respectively [16]. The Large T Antigen (LTAg) protein is encoded by the BKPyVgp5 gene locus located in the early region of the BKPy virus genome. Also, LTAg transforms both human and animal cells. In fact, LTAg interferes with the pRB and p53 families' functions as tumor suppressors [20]. Considering the possible carcinogenic role of the BKV in some malignancies and as the BKV could establish long-term latency infection in the urinary-genital tract, we performed a case-control study to assess the existence of the BKPyVgp5 gene in malignant and non-cancerous (BPH) prostate tissue samples.

## MATERIALS AND METHODS

**Sample Design: **In this cross-sectional study, 98 formalin-fixed paraffin-embedded (FFPE) prostate tissue samples, including 49 cancerous tissue samples (case group) and 49 benign prostatic hyperplasia samples (control group), were gathered. The tissue block samples were randomly selected from the archive of the pathology department of a tertiary care university hospital in Mashhad, Iran, from 2009 to 2019. Age and Gleason scores (for PCa patients) were also recorded. The Gleason score is a routine grading system to evaluate the aggressiveness of prostate cancer based on its microscopic appearance. It is determined by examining the histology and cellular composition of the tissue sample. The scoring system is mainly based on architecture and cellular characteristics of the tumor cells. An expert pathologist confirmed a PCa or BPH tissue diagnosis and determined the Gleason score according to the Gleason grading system [21]. A lower Gleason score indicates a less aggressive form of PCa, whereas a higher score suggests more aggressive cancer. The Medical Ethics Committee of Mashhad University of Medical Sciences approved the study (IR.MUMS.MEDICAL.REC.1398.318).

**Deparaffinization and DNA Extraction: **The prostate tissue blocks were cut into roughly seven 8-μm slices using a microtome. The tissue sections were collected inside a sterile 1.5 ml Eppendorf tube with minimal paraffin entry. Deparaffinization and dehydration were performed with xylene and ethanol. First, 1 ml of xylene was added to microtubes containing 25 mg of paraffin tissue. After the rigorous vortex, the tubes were incubated at 56°C for 30 minutes. Afterward, the tubes underwent centrifugation at 14,000 rpm for 1 minute, and the supernatant liquid was removed. Then, 1 ml of 96% ethanol was added to the microtubes, and after incubation at 56°C for 30 minutes, the tubes were centrifuged at 14,000 rpm for 3 minutes, and the supernatant was removed. Paraffin was removed from the tissue samples by repeating this step twice or thrice. Finally, the samples were placed on a heat block to evaporate the remaining ethanol. Eventually, Tissue DNA Extraction Micro Kit (Favorgen Biotech, Pingtung, Taiwan) was used to extract DNA per the manufacturer's instructions. Lysing and digestion of prostate tissue samples' cells were conducted by FATG1 buffer (200 µl), and proteinase K (20 µl), and the mixture was thoroughly vortexed. The mixture was incubated at 60°C for 60 minutes until the tissue was lysed completely. Next, 200 µl of FATG2 buffer and 200 µl of ethanol 96% were added to the sample mixture and mixed thoroughly by pulse vortexing. Afterward, the mixture was transferred to a mini-column and placed in a 2 ml collection tube. The columns were centrifuged for 1 minute and washed twice with a wash buffer. The extracted DNA was eluted from the mini-columns with an elution buffer in the final extraction step. To evaluate the quality and concentration of the extracted DNA, a nanodrop spectrophotometer provided by Thermo Fisher Scientific was utilized. The resulting DNA was subsequently stored at -20°C to ensure its preservation for future use.

**Nested PCR for** **BKPyVgp5) LTAg): **A nested PCR was used to identify the BKPyVgp5 genome for all 96 tissue samples after DNA extraction. The internal and external primers were used as previously described [22]. The outer primer pair (JCVF1) 5^’^-CTGGGTTAAAGTCATG CT-3^’^ and (JCVR1) 5^’^-GGTAGAAGACCCTAAAGACT-3^’^ and the inner primer pair (BKVF2) 5^’_^AAGTATTCCTTATTCACACC-3^’^ and (BKVR2) 5^’^-CCCTCTGATCTACACCAG-3^’^ were used to amplify the 385-bp and 278-bp fragments, respectively. The first and second rounds of the PCR amplification were carried out in a total volume of 20 µL, including 10 µL of Taq DNA polymerase 2X master mix red (Ampliqon, Denmark), containing Tris-HCl pH=8.5, (NH_4_)_2_SO_4_, 4 mM MgCl_2_, 0.2% tween 20, 0.4 mM dNTPs, 0.2 units/μl Ampliqon Taq DNA polymerase inert red dye and stabilizer, 5 µL of template DNA, 1 µL of each primer (5 pmol/μl), and 3 μl of nuclease-free water. In FlexCycler (Analytik Jena, Jena, Germany), the first round of the PCR thermal cycling program included initial denaturation at 94°C for 2 minutes, followed by 35 cycles of denaturation at 94°C for 30 seconds, annealing at 48°C for 30 seconds, extension at 72°C for 30 seconds, and a final extension for 5 minutes. For the second round of nested PCR, 5 µL of the first-round PCR product was used. The second round protocol was as follows: Initial denaturation at 94°C for 2 minutes, followed by 35 cycles at 94°C for 30 seconds, annealing at 45°C for 30 seconds, and extension at 72°C for 30 seconds and a final extension at the same temperature for 5 minutes. Finally, the nested-PCR products were electrophoresed on a 1.5% agarose gel and visualized under UV by a safe Nucleic Acid Stain (DNA Green Viewer™, Parstous, Iran). A positive control provided by Dr. Meshkat was used in all experiments. 

**Statistical Analysis: **The data analysis was performed by the Statistical Package for the Social Science version 18 (SPSS v.18.0.0 ). Qualitative variables were described by frequency and percentage, and quantitative variables were described by mean and standard deviation. The quantitative variables were compared between the two groups by the Student *t*-test. The relationship of qualitative variables was assessed by the Fisher exact test. The presence of LTAg as a risk factor among patients was evaluated using logistic regression analysis using the "Enter" method and reported as a 95% confidence interval for the Odds Ratio (OR). The goodness of fit in this regression was checked by Cox & Snell R-square. All tests were two-tailed, and a p value below 0.05 was considered statistically significant.

## RESULTS AND DISCUSSION

**Figure 1:**
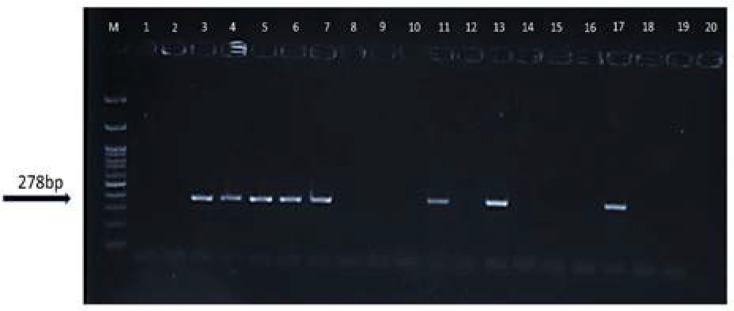
Detection of the BKPyVgp5 gene (LTAg of BKV) by nested-PCR from the extracted genome

In this study, the mean age of PCa and BPH patients was 73.4 ± 10.5 and 69.9 ± 10.1 years, respectively. There was no significant difference in age between the PCa and BPH groups (p = 0.091). Based on cellular examination, prostate cancer could be scored from 2 to 10. Our patients ranged from 6 to 10, indicating more aggressive tumors with a higher mortality rate. The Gleason score was available for 40 patients, with the most patients having a score of 7 (16 patients) and the least patients having a score of 6 (2 patients) and 10 (2 patients). The highest frequency of BKV genome was detected in PCa with a Gleason score of 9 (41.6%), followed by a score of 7 (33.3%), 8 (20.8%), and 6 (4.2%). The Gleason score of PCa is summarized in Table 2. A 278-bp fragment of the BKPyVgp5 genome (LTAg) was found by nested PCR using internal primers in 33.7% (33/98) of all tissue samples, including 53.1% (26/49) of PCa and 14.3% (7/49) of BPH (p<0.001) (Table 1, Fig. 1). Logistic regression showed that the presence of LTAg could increase the odds of PCa by a factor of 6.78 (95% CI: 2.55-18.02). The goodness of fit of this model based on the Cox & Snell R-square was 16.2%.

**Table 1:** Frequency of BKPyVgp5 gene (LTAg) among PCa and BPH groups

**BKPyVgp5 gene**	**No.(%)**	**PCa patients **	**BPH patients **	**p-value**
**Positive**	33(33.7)	26 (53.1)	7 (14.3)	<0.001*
**Negative**	65 (66.3)	23 (46.9)	42 (85.7)	

*Fisher exact test

**Table 2:** Frequency of BKPyVgp5 gene in different classes of Gleason Score

**BKPyVgp5 gene**	**Gleason Score**					**Total**
	**6**	**7**	**8**	**9**	**10**	
**Positive**	1 (4.2%)	8 (33.3%)	5 (20.8%)	10 (41.6%)	0 (0.0%)	24
**Negative**	1(6.25%)	8 (50.0%)	0 (0.0%)	5 (31.2%)	2 (12.5%)	16
**Total**	2	16	5	15	2	40

The figure shows a representative of the sample. M: DNA marker, 10: negative control, 11: positive control, Lans 1, 2, 8, 9, 12, 14, 15, 16, 18, 19, and 20: negative samples, 3-7, 13, and 17: positive samples.

Prostate cancer (PCa) is the third most common cancer according to the GLOBOCAN 2020 estimate [23]. Several studies suggest that viral infection-induced inflammation may play a role in PCa [7]. BKV stimulates the expression of large T antigen and small T antigen, inactivates tumor suppressor proteins such as p53 and pRB, and disrupts cell cycle control [24].

This study investigated the prevalence of the BKPyVgp5 (LTAg) gene in cancerous and non-cancerous prostate tissue samples. Our results indicated significant differences in the presence of the BKPyVgp5 (LTAg) genome between the PCa and BPH samples (P<0.001), consistent with other studies [25-27]. Contrary to our study, some studies [28, 29] found no significant differences in the presence of BKV DNA between PCa and BPH patients. Differences in sample size, analyte detection methods, sample types, and prevalence of this virus in different regions may explain the variation in study results.

In our study, the OR of developing PCa in the presence of the BKPyVgp5 genome was 6.78, which means that patients with the LTAg genome had about seven times higher risk of developing PCa than those without this genome. This is supported by other studies [25, 27, 30], which indicated that the presence of BKV, especially LTAg, could increase the risk of developing prostate cancer.

The Gleason score of 40 PCa patients was available, ranging from 6 to 10. Although there was no relationship between the Gleason score and the presence of the BKPyVgp5 gene, most PCa patients who were positive for the LTAg gene of BKV were observed among paraffin-embedded tissue blocks with a Gleason score of 9. The results of the study by Gorish et al. are consistent with our findings [26]. In their study, the highest BKV LTAg prevalence was reported in PCa patients with Gleason scores of 7 and 9. Also, a Gleason score of 10 had the lowest BKV LTAg prevalence.

In conclusion, PCa patients were significantly more likely to carry the BKPyVgp5 gene than BPH patients. Finally, in this study, we tried to clarify the association of BK virus with prostate cancer. However, investigation of the carcinogenic mechanisms of other members of the polyomavirus family is recommended for future studies using in vitro and in vivo molecular methods.

## Conflict of Interest:

The authors declare no conflict of interest.
